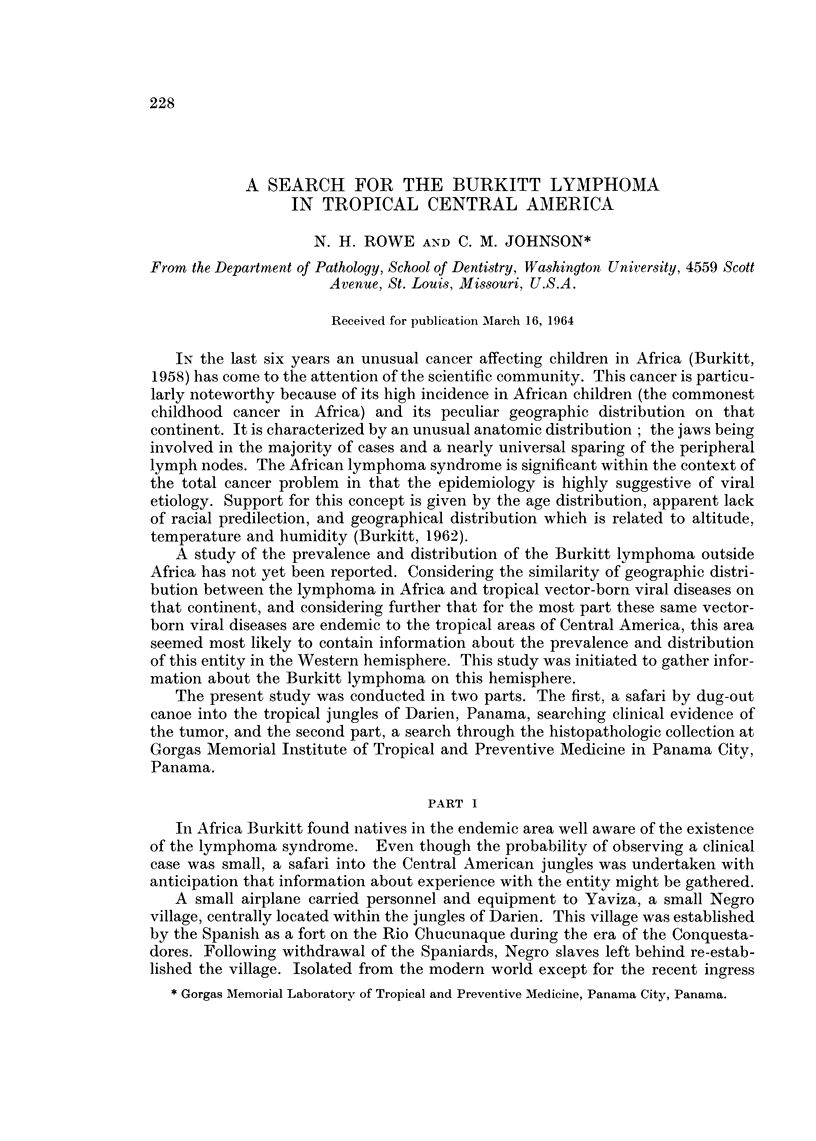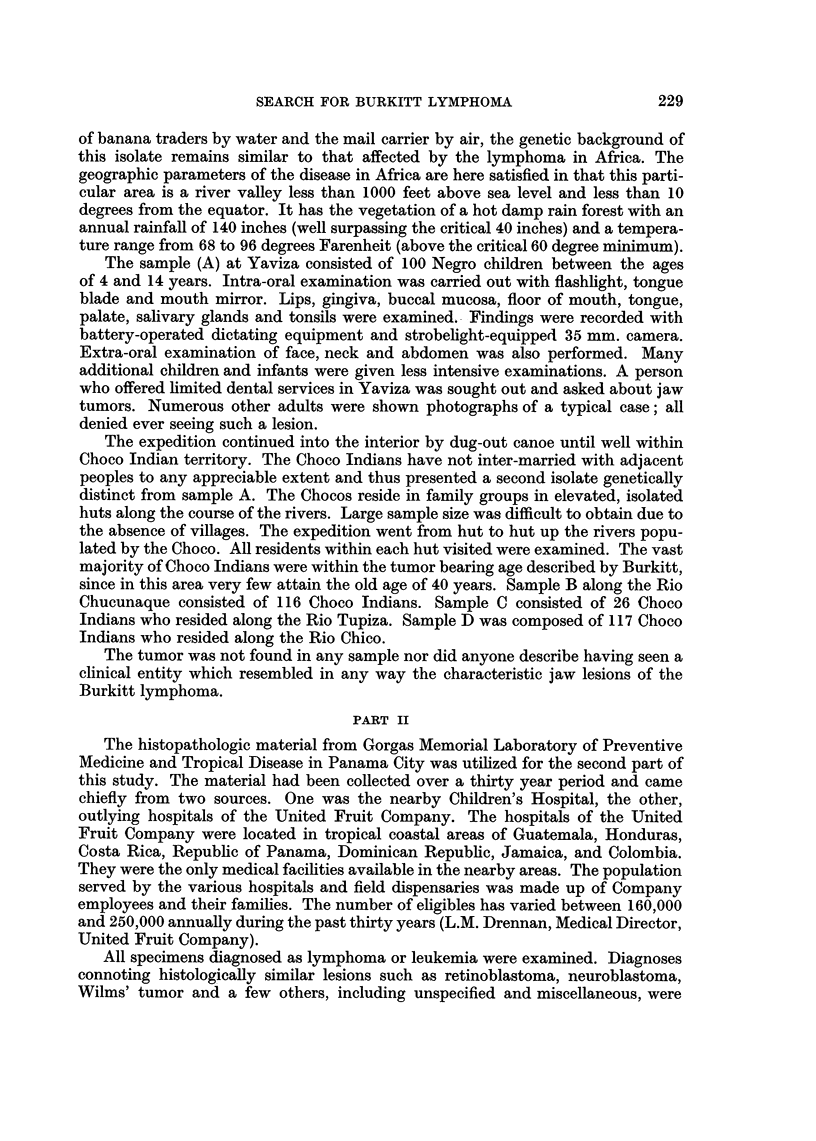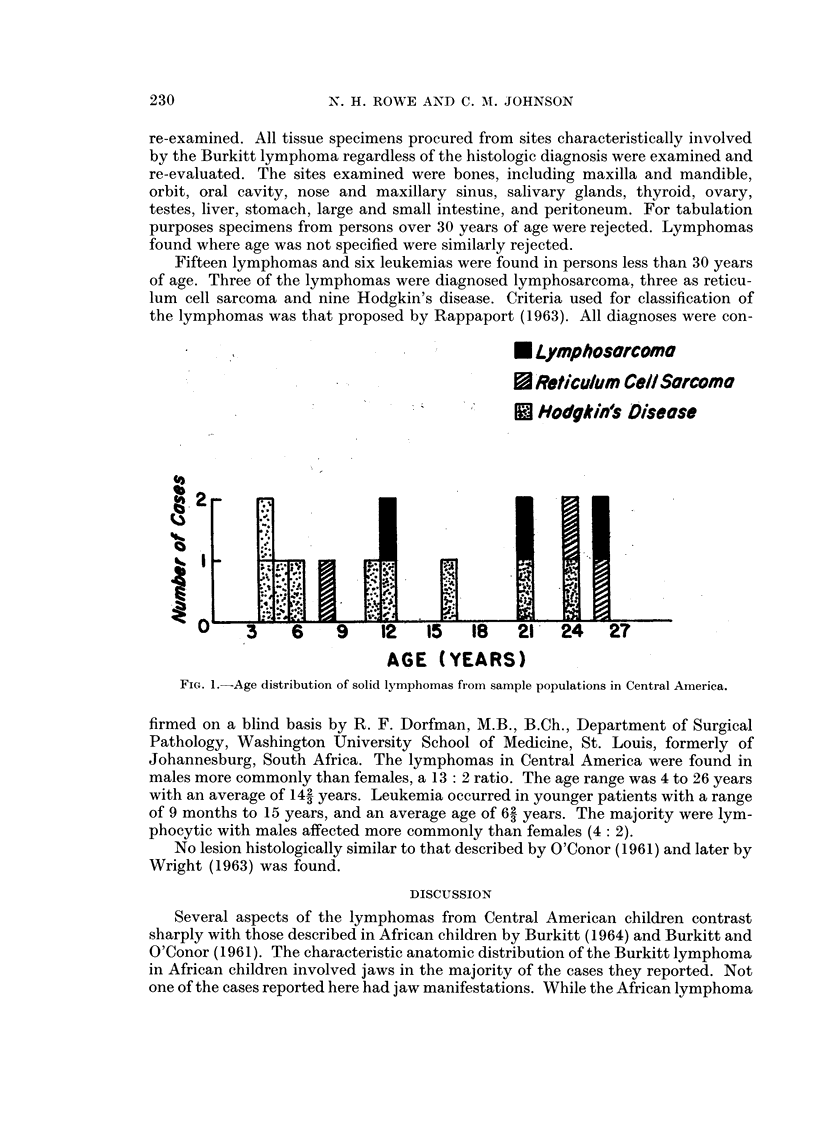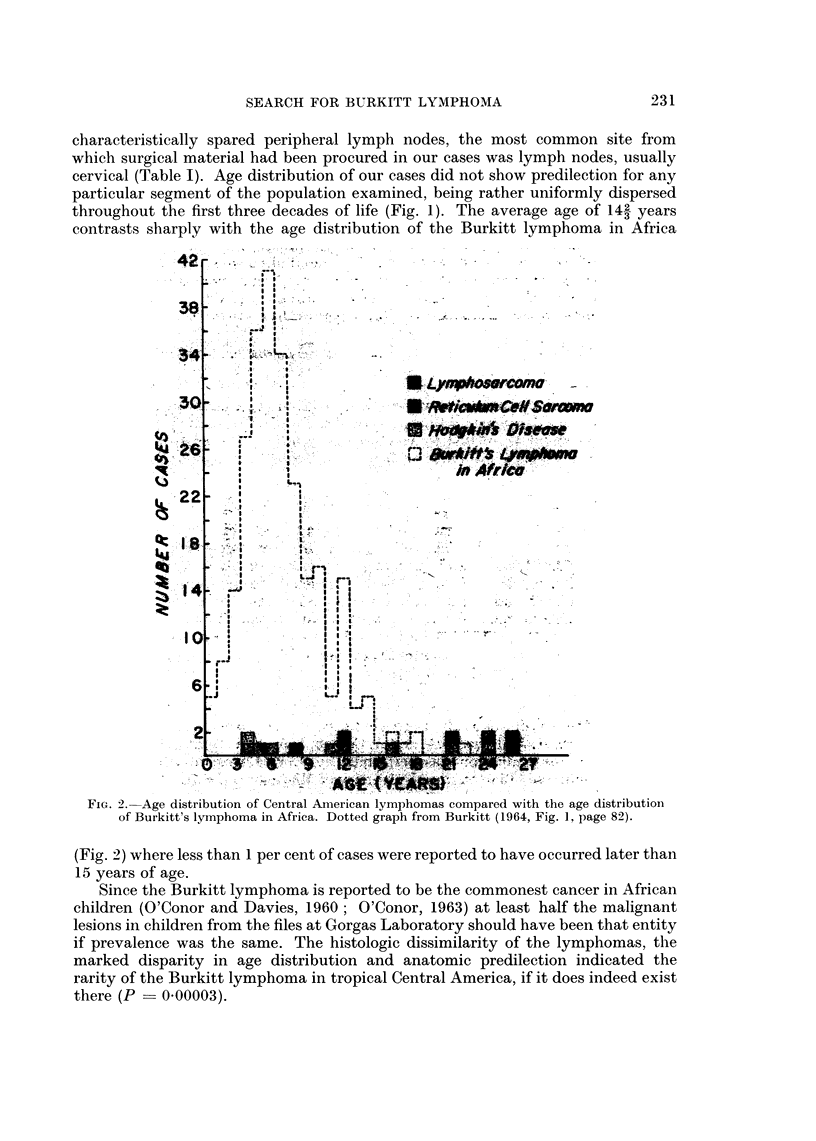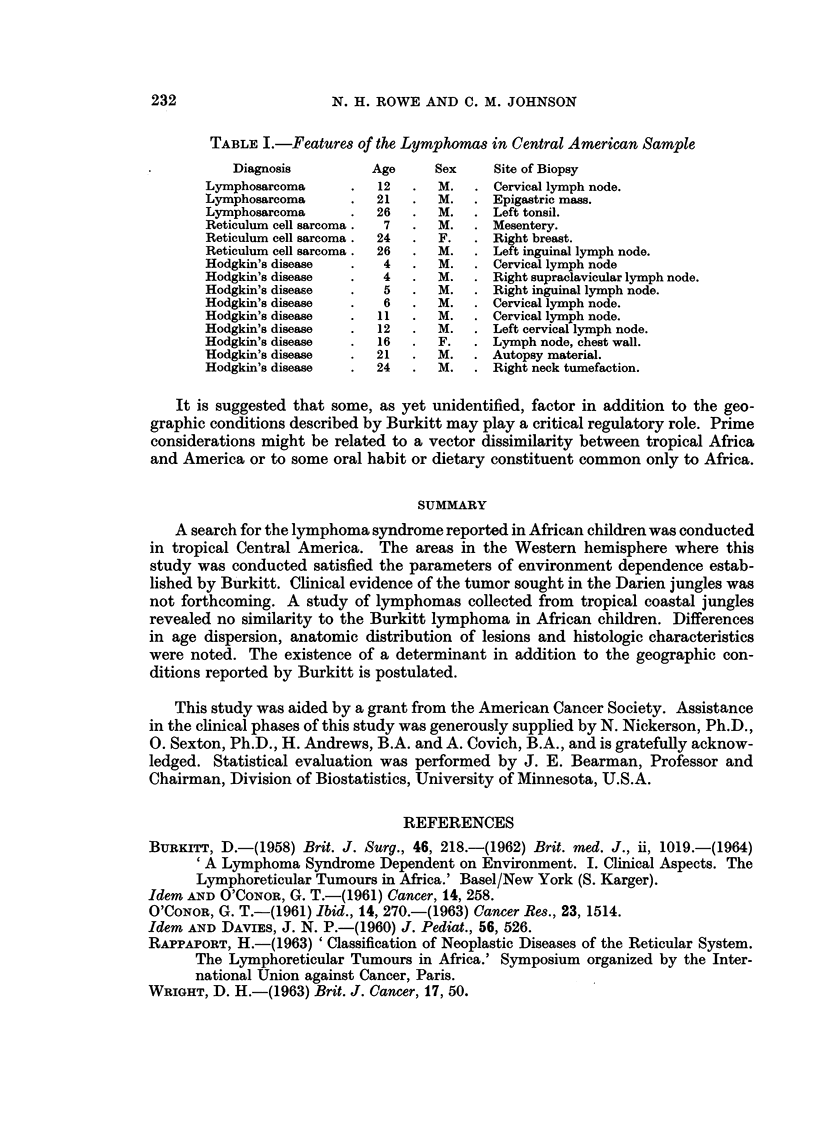# A Search for the Burkitt Lymphoma in Tropical Central America

**DOI:** 10.1038/bjc.1964.24

**Published:** 1964-06

**Authors:** N. H. Rowe, C. M. Johnson


### A SEARCH FOR THE BURKITT LYMPHOMA IN TROPICAL CENTRAL AMERICA

N. H. ROWE AND C. M. JOHNSON*

*From the Department of Pathology, School of Dentistry, Washington University,* 4559 *Scott Avenue, St. Louis, Missouri, U.S.A.*

Received for publication March 16, 1964

IN the last six years an unusual cancer affecting children in Africa (Burkitt, 1958) has come to the attention of the scientific community. This cancer is particularly noteworthy because of its high incidence in African children (the commonest childhood cancer in Africa) and its peculiar geographic distribution on that continent. It is characterized by an unusual anatomic distribution ; the jaws being involved in the majority of cases and a nearly universal sparing of the peripheral lymph nodes. The African lymphoma syndrome is significant within the context of the total cancer problem in that the epidemiology is highly suggestive of viral etiology. Support for this concept is given by the age distribution, apparent lack of racial predilection, and geographical distribution which is related to altitude, temperature and humidity (Burkitt, 1962).

A study of the prevalence and distribution of the Burkitt lymphoma outside Africa has not yet been reported. Considering the similarity of geographic distribution between the lymphoma in Africa and tropical vector-born viral diseases on that continent, and considering further that for the most part these same vector-born viral diseases are endemic to the tropical areas of Central America, this area seemed most likely to contain information about the prevalence and distribution of this entity in the Western hemisphere. This study was initiated to gather information about the Burkitt lymphoma on this hemisphere.

The present study was conducted in two parts. The first, a safari by dug-out canoe into the tropical jungles of Darien, Panama, searching clinical evidence of the tumor, and the second part, a search through the histopathologic collection at Gorgas Memorial Institute of Tropical and Preventive Medicine in Panama City, Panama.

### PART I

In Africa Burkitt found natives in the endemic area well aware of the existence of the lymphoma syndrome. Even though the probability of observing a clinical case was small, a safari into the Central American jungles was undertaken with anticipation that information about experience with the entity might be gathered.

A small airplane carried personnel and equipment to Yaviza, a small Negro village, centrally located within the jungles of Darien. This village was established by the Spanish as a fort on the Rio Chucunaque during the era of the Conquestadores. Following withdrawal of the Spaniards, Negro slaves left behind re-established the village. Isolated from the modern world except for the recent ingress

* Gorgas Memorial Laboratory of Tropical and Preventive Medicine, Panama City, Panama.

of banana traders by water and the mail carrier by air, the genetic background of this isolate remains similar to that affected by the lymphoma in Africa. The geographic parameters of the disease in Africa are here satisfied in that this particular area is a river valley less than 1000 feet above sea level and less than 10 degrees from the equator. It has the vegetation of a hot damp rain forest with an annual rainfall of 140 inches (well surpassing the critical 40 inches) and a temperature range from 68 to 96 degrees Farenheit (above the critical 60 degree minimum).

The sample (A) at Yaviza consisted of 100 Negro children between the ages of 4 and 14 years. Intra-oral examination was carried out with flashlight, tongue blade and mouth mirror. Lips, gingiva, buccal mucosa, floor of mouth, tongue, palate, salivary glands and tonsils were examined. Findings were recorded with battery-operated dictating equipment and strobelight-equipped 35 mm. camera. Extra-oral examination of face, neck and abdomen was also performed. Many additional children and infants were given less intensive examinations. A person who offered limited dental services in Yaviza was sought out and asked about jaw tumors. Numerous other adults were shown photographs of a typical case; all denied ever seeing such a lesion.

The expedition continued into the interior by dug-out canoe until well within Choco Indian territory. The Choco Indians have not inter-married with adjacent peoples to any appreciable extent and thus presented a second isolate genetically distinct from sample A. The Chocos reside in family groups in elevated, isolated huts along the course of the rivers. Large sample size was difficult to obtain due to the absence of villages. The expedition went from hut to hut up the rivers populated by the Choco. All residents within each hut visited were examined. The vast majority of Choco Indians were within the tumor bearing age described by Burkitt, since in this area very few attain the old age of 40 years. Sample B along the Rio Chucunaque consisted of 116 Choco Indians. Sample C consisted of 26 Choco Indians who resided along the Rio Tupiza. Sample D was composed of 117 Choco Indians who resided along the Rio Chico.

The tumor was not found in any sample nor did anyone describe having seen a clinical entity which resembled in any way the characteristic jaw lesions of the Burkitt lymphoma.

### PART II

The histopathologic material from Gorgas Memorial Laboratory of Preventive Medicine and Tropical Disease in Panama City was utilized for the second part of this study. The material had been collected over a thirty year period and came chiefly from two sources. One was the nearby Children's Hospital, the other, outlying hospitals of the United Fruit Company. The hospitals of the United Fruit Company were located in tropical coastal areas of Guatemala, Honduras, Costa Rica, Republic of Panama, Dominican Republic, Jamaica, and Colombia. They were the only medical facilities available in the nearby areas. The population served by the various hospitals and field dispensaries was made up of Company employees and their families. The number of eligibles has varied between 160,000 and 250,000 annually during the past thirty years (L.M. Drennan, Medical Director, United Fruit Company).

All specimens diagnosed as lymphoma or leukemia were examined. Diagnoses connoting histologically similar lesions such as retinoblastoma, neuroblastoma, Wilms' tumor and a few others, including unspecified and miscellaneous, were re-examined. All tissue specimens procured from sites characteristically involved by the Burkitt lymphoma regardless of the histologic diagnosis were examined and re-evaluated. The sites examined were bones, including maxilla and mandible, orbit, oral cavity, nose and maxillary sinus, salivary glands, thyroid, ovary, testes, liver, stomach, large and small intestine, and peritoneum. For tabulation purposes specimens from persons over 30 years of age were rejected. Lymphomas found where age was not specified were similarly rejected.

Fifteen lymphomas and six leukemias were found in persons less than 30 years of age. Three of the lymphomas were diagnosed lymphosarcoma, three as reticulum cell sarcoma and nine Hodgkin's disease. Criteria used for classification of the lymphomas was that proposed by Rappaport (1963). All diagnoses were con-

FIG. 1.—Age distribution of solid lymphomas from sample populations in Central America.

firmed on a blind basis by R. F. Dorfman, M.B., B.Ch., Department of Surgical Pathology, Washington University School of Medicine, St. Louis, formerly of Johannesburg, South Africa. The lymphomas in Central America were found in males more commonly than females, a 13 : 2 ratio. The age range was 4 to 26 years with an average of $14\frac{2}{3}$ years. Leukemia occurred in younger patients with a range of 9 months to 15 years, and an average age of $6\frac{2}{3}$ years. The majority were lymphocytic with males affected more commonly than females (4 : 2).

No lesion histologically similar to that described by O'Conor (1961) and later by Wright (1963) was found.

### DISCUSSION

Several aspects of the lymphomas from Central American children contrast sharply with those described in African children by Burkitt (1964) and Burkitt and O'Conor (1961). The characteristic anatomic distribution of the Burkitt lymphoma in African children involved jaws in the majority of the cases they reported. Not one of the cases reported here had jaw manifestations. While the African lymphoma characteristically spared peripheral lymph nodes, the most common site from which surgical material had been procured in our cases was lymph nodes, usually cervical (Table I). Age distribution of our cases did not show predilection for any particular segment of the population examined, being rather uniformly dispersed throughout the first three decades of life (Fig. 1). The average age of $14\frac{2}{3}$ years contrasts sharply with the age distribution of the Burkitt lymphoma in Africa

FIG. 2.—Age distribution of Central American lymphomas compared with the age distribution of Burkitt's lymphoma in Africa. Dotted graph from Burkitt (1964, Fig. 1, page 82).

(Fig. 2) where less than 1 per cent of cases were reported to have occurred later than 15 years of age.

Since the Burkitt lymphoma is reported to be the commonest cancer in African children (O'Conor and Davies, 1960 ; O'Conor, 1963) at least half the malignant lesions in children from the files at Gorgas Laboratory should have been that entity if prevalence was the same. The histologic dissimilarity of the lymphomas, the marked disparity in age distribution and anatomic predilection indicated the rarity of the Burkitt lymphoma in tropical Central America, if it does indeed exist there ($P = 0{\cdot}00003$).

TABLE I.—*Features of the Lymphomas in Central American Sample*

| Diagnosis | Age | Sex | Site of Biopsy |
|---|---|---|---|
| Lymphosarcoma | 12 | M. | Cervical lymph node. |
| Lymphosarcoma | 21 | M. | Epigastric mass. |
| Lymphosarcoma | 26 | M. | Left tonsil. |
| Reticulum cell sarcoma | 7 | M. | Mesentery. |
| Reticulum cell sarcoma | 24 | F. | Right breast. |
| Reticulum cell sarcoma | 26 | M. | Left inguinal lymph node. |
| Hodgkin's disease | 4 | M. | Cervical lymph node |
| Hodgkin's disease | 4 | M. | Right supraclavicular lymph node. |
| Hodgkin's disease | 5 | M. | Right inguinal lymph node. |
| Hodgkin's disease | 6 | M. | Cervical lymph node. |
| Hodgkin's disease | 11 | M. | Cervical lymph node. |
| Hodgkin's disease | 12 | M. | Left cervical lymph node. |
| Hodgkin's disease | 16 | F. | Lymph node, chest wall. |
| Hodgkin's disease | 21 | M. | Autopsy material. |
| Hodgkin's disease | 24 | M. | Right neck tumefaction. |

It is suggested that some, as yet unidentified, factor in addition to the geographic conditions described by Burkitt may play a critical regulatory role. Prime considerations might be related to a vector dissimilarity between tropical Africa and America or to some oral habit or dietary constituent common only to Africa.

SUMMARY

A search for the lymphoma syndrome reported in African children was conducted in tropical Central America. The areas in the Western hemisphere where this study was conducted satisfied the parameters of environment dependence established by Burkitt. Clinical evidence of the tumor sought in the Darien jungles was not forthcoming. A study of lymphomas collected from tropical coastal jungles revealed no similarity to the Burkitt lymphoma in African children. Differences in age dispersion, anatomic distribution of lesions and histologic characteristics were noted. The existence of a determinant in addition to the geographic conditions reported by Burkitt is postulated.

This study was aided by a grant from the American Cancer Society. Assistance in the clinical phases of this study was generously supplied by N. Nickerson, Ph.D., O. Sexton, Ph.D., H. Andrews, B.A. and A. Covich, B.A., and is gratefully acknowledged. Statistical evaluation was performed by J. E. Bearman, Professor and Chairman, Division of Biostatistics, University of Minnesota, U.S.A.

### REFERENCES

BURKITT, D.—(1958) *Brit. J. Surg.*, **46**, 218.—(1962) *Brit. med. J.*, ii, 1019.—(1964) 'A Lymphoma Syndrome Dependent on Environment. I. Clinical Aspects. The Lymphoreticular Tumours in Africa.' Basel/New York (S. Karger).

*Idem* AND O'CONOR, G. T.—(1961) *Cancer*, **14**, 258.

O'CONOR, G. T.—(1961) *Ibid.*, **14**, 270.—(1963) *Cancer Res.*, **23**, 1514.

*Idem* AND DAVIES, J. N. P.—(1960) *J. Pediat.*, **56**, 526.

RAPPAPORT, H.—(1963) 'Classification of Neoplastic Diseases of the Reticular System. The Lymphoreticular Tumours in Africa.' Symposium organized by the International Union against Cancer, Paris.

WRIGHT, D. H.—(1963) *Brit. J. Cancer*, **17**, 50.